# Cranial thoracic myelopathies (T1-T6 vertebrae): Retrospective evaluation of the signalment, clinical presentation, and, presumptive or final diagnoses in 84 dogs

**DOI:** 10.3389/fvets.2022.960912

**Published:** 2022-09-12

**Authors:** Bruno A. Lopes, Edward J. Ives, Roberto José-López, Rodrigo Gutierrez-Quintana, Jad Abouzeid, Paul Freeman, José Ignacio Redondo, Daniel Sánchez-Masián

**Affiliations:** ^1^Anderson Moores Veterinary Specialists, Part of Linnaeus Veterinary Limited, Hursley, United Kingdom; ^2^School of Veterinary Medicine, College of Medical, Veterinary and Life Sciences, University of Glasgow, Glasgow, United Kingdom; ^3^Department of Veterinary Medicine, University of Cambridge, Cambridge, United Kingdom; ^4^Departamento de Medicina y Cirugía Animal, Facultad de Veterinaria, Universidad Cardenal Herrera-CEU, CEU Universities, Valencia, Spain

**Keywords:** advanced imaging, ataxia, canine, neoplasia, neurological, spinal cord, two-engine gait

## Abstract

The aim of the study was to describe the signalment, clinical presentation and presumptive or final diagnoses of dogs with cranial thoracic spinal cord lesions identified on advanced imaging. Retrospective evaluation of the databases of three veterinary specialty centres, between 2009 and 2021, was performed to identify dogs with a lesion affecting the cranial thoracic vertebral column (T1-T6 vertebrae) as the primary cause for presenting signs of myelopathy and/or spinal pain. Eighty-four dogs were included in the study, with the majority (*n* = 76) presenting with a progressive history of over 4-weeks' duration. On neurologic examination, most dogs were ambulatory (*n* = 64), and the most common neuroanatomic localisation was the T3-L3 spinal cord segments (*n* = 63). Twelve dogs (14%) showed a short-strided thoracic limb gait on clinical examination. The most common diagnosis was neoplasia (*n* = 33), followed by anomalies (*n* = 22, including vertebral body malformations in 14 dogs) and degenerative disorders (*n* = 16, with intervertebral disc protrusion diagnosed in 9 dogs). The most common vertebrae affected were T3 and T5. Most dogs with degenerative conditions showed asymmetric clinical signs, and the majority of dogs with neoplasia showed signs of spinal hyperaesthesia on examination. The findings of this study describe the clinical signs and presumptive or final diagnoses associated with lesions affecting the cranial thoracic spinal cord. When combined with the signalment and clinical history, this information can assist in both the recognition of and problem-based approach to these cases.

## Introduction

Disorders that affect the spinal cord or vertebral column, leading to pain and/or neurologic deficits, are a common occurrence in small animal practise. Performing a thorough neurologic examination in order to determine an accurate neuroanatomic localisation is vital in these cases, as the differential diagnoses and subsequent diagnostic tests are dependent upon this information (1). For the neuroanatomic localisation of disorders affecting the spinal cord, termed myelopathies, the individual spinal cord segments are divided into four broad regions based on the expected clinical presentation associated with a lesion affecting these regions: cranial cervical (C1-C5), cervicothoracic (C6-T2), thoracolumbar (T3-L3) and lumbosacral (L4-S3) (1). However, there is not a direct correlation between the location of these spinal cord segments and the vertebrae with the same name. With the exception of the last two thoracic and first two (occasionally three) lumbar spinal cord segments, the majority of spinal cord segments reside within the vertebra(e) cranial to those of the same number (2). Thus, the T3-L3 spinal cord segments are typically located within the T2 to L3 vertebral bodies in dogs (2, 3).

Lesions affecting the cranial thoracic vertebral column, classified in this article as between the T1 and T6 vertebrae, will therefore most commonly result in clinical signs referable to dysfunction of the T3-L3 spinal cord segments. These signs may include pelvic limb ataxia, upper motor neuron paraparesis/paraplegia, intact pelvic limb spinal reflexes, interruption of the cutaneous trunci muscle reflex (CTMR), urinary and/or faecal incontinence, variable discomfort and, in severe cases, anaesthesia caudal to the lesion (3). A cranial thoracic lesion may be particularly suspected if, in addition to the aforementioned T3-L3 signs, there is an absent CTMR or interruption of this reflex cranial to the mid thoracic region, truncal ataxia and weakness, potentially resulting in an inability to rise into a sitting position, evidence of focal pain upon palpation of the cranial thoracic vertebral column, or Horner syndrome if the lesion is affecting the T1-T3 spinal cord segments and/or respective nerve roots (1–3). Finally, a short-strided thoracic limb gait in conjunction with a long-strided ataxic pelvic limb gait, resulting in a “two-engine” or “disconnected” gait (3–5), has also been anecdotally reported in animals with lesions affecting the cranial thoracic vertebral column, as far caudal as the T5 vertebra (4).

When considering the differential diagnosis for a T3-L3 myelopathy, a subdivision of these segments into cranial-mid thoracic and thoracolumbar areas has been suggested (1). Intervertebral disc disease is the most common disorder affecting the spinal cord in dogs and most frequently occurs around the thoracolumbar junction and lumbar vertebral column (1, 3). However, it is considered rare in the cranial thoracic region, due to the presence of the intercapital ligaments that connect the rib heads between T2-T10, which are believed to increase the dorsal constraint of the intervertebral disc and make clinically significant disc disease less likely (1, 6). Therefore, it has been suggested that alternative differential diagnoses should be considered in dogs when a cranial thoracic spinal cord lesion is suspected on examination, such as neoplasia, discospondylitis, and congenital vertebral malformations (1).

The prevalence and type of disorders affecting the cranial thoracic vertebral column in dogs has been scarcely described in the veterinary literature. These are limited to case reports, small case series or sporadic cases within larger studies describing intervertebral disc disease (7–17), calcinosis circumscripta (18, 19), congenital malformations [cranial thoracic vertebral canal stenosis (20–23), thoracic vertebral body malformations (24–28), spinal arachnoid diverticula (29), spina bifida ± other associated abnormalities (30–33)], neoplasia [chondrosarcoma (34), meningioma (13, 35), multiple myeloma (36), myxosarcoma (37), nerve sheath tumour (35)], discospondylitis (14, 38) or fracture/luxation (14, 39). To the authors' knowledge, there are no studies dedicated to the description of the signalment, clinical signs and clinical prevalence of the different disease processes affecting this particular region of the vertebral column. This information would be useful in clinical practise, as it would inform the clinician of the most likely differential diagnoses to consider in an animal of a particular signalment that presents with neurological deficits consistent with a lesion affecting the cranial thoracic region.

The primary goal of this study was to describe the signalment, clinical presentation and presumptive or final diagnoses in animals that presented for clinical signs referable to lesions affecting the cranial thoracic spinal cord (T1-T6 vertebrae) identified on advanced imaging. A secondary goal consisted in evaluating particular clinical features, including disease onset, progression, presence of apparent discomfort, and frequency of an apparent “two-engine” gait in cases with such lesions.

## Materials and methods

This retrospective study was approved by the University of Glasgow School of Veterinary Medicine Research Ethics Committee (EA07/21). The medical records and imaging databases of three veterinary referral centres (Anderson Moores Veterinary Specialists, Queen's Veterinary School Hospital—Cambridge University, and University of Glasgow Small Animal Hospital), for the period between 2009 and 2021, were retrospectively searched for dogs with lesions affecting the cranial thoracic vertebral column (T1-T6). Only lesions identified using advanced imaging [magnetic resonance imaging (MRI) and/or computed tomography (CT)] and that were determined to be the primary cause for the presenting clinical signs were included in the study. Information retrieved from the medical records included breed, sex, neuter status, weight, age at diagnosis, onset, progression, duration of clinical signs, general physical examination findings, and neurologic examination findings, including neuroanatomic localisation (clinical localisation according to affected spinal cord segments), ambulation status, CTMR abnormalities, asymmetry of the clinical signs, and presence/absence of the following: urinary/faecal incontinence, Horner syndrome, apparent discomfort on vertebral column palpation and short-strided thoracic limb gait. The imaging modality used, specific imaging findings, affected site(s) referable to vertebral number (rather than spinal cord segment), lesion characteristics and location pattern, results of other diagnostic tests performed, the presumptive diagnosis and final diagnosis (if achieved) were also evaluated. Characterisation of the rate of onset was limited to the information available following a retrospective review of the medical history and was therefore defined as “acute” or “chronic” based on the description or terminology used in the clinical records rather than use of a pre-defined distinction. The duration of clinical signs prior to referral (in days) was recorded and divided into 3 categories: “ <2 weeks,” “2–4 weeks,” or “over 4 weeks”. Disease progression was defined as progressive, static, improving or episodic (with a return to normality between the occurrence of signs), based upon the clinical history recorded.

All the imaging studies were performed under sedation or general anaesthesia using either MRI (Anderson Moores Veterinary Specialists: 1.5-Tesla Philips Gyroscan Intera, 1.5-Tesla Philips Achieva and 1.5-Tesla Philips Intera Achieva, Koninklijke Philips N.V., Amsterdam, Netherlands; Queen's Veterinary School Hospital—Cambridge University: 0.27-Tesla Esaote VetMR Grande, Genova, Italy; University of Glasgow Small Animal Hospital: 1.5-Tesla Siemens Magnetom Essenza, Erlangen, Germany) or a CT (Anderson Moores Veterinary Specialists: Somaton Perspective 64-Slice, Siemens, Erlangen, Germany; University of Glasgow Small Animal Hospital: Somaton Spirit Dual Slice, Siemens, Erlangen, Germany), with the range of scanners dependent on the centre and date performed. The MRI sequences acquired varied between cases, in accordance with the clinician's preference and/or different centre-related protocols but included a minimum of a T2-weighted (T2w) sequence in the sagittal and transverse planes, and a T1-weighted (T1w) sequence in the transverse plane. Contrast agent was used as per the clinician's preference, with gadolinium-based contrast [Gadovist (Gadobutrol) 1.0 mmol/mL, Bayer PLC] for MRI studies and non-ionic iodinated contrast [Omnipaque (Iohexol) 300 mg iodine/mL, GE Healthcare Ireland, Cork, Ireland] for CT studies.

All imaging studies were retrospectively reviewed by a veterinary neurology resident in training (BL) and a Board-Certified specialist in veterinary neurology (DSM). Lesion location pattern was described as intramedullary, intradural-extramedullary, or extradural based on the imaging features. The affected site was recorded based on the previous imaging report and after review of all imaging studies. The final diagnoses were grouped based on their definitive or presumptive disease mechanism, as described elsewhere (1), using the VITAMIN-D mnemonic (vascular, inflammatory, infectious, trauma, toxicity, anomalous, metabolic, idiopathic, neoplasia, and degenerative). Dogs of any age, breed, gender or neuter status, with one or more lesions identified on advanced imaging in the region of the T1–T6 vertebrae considered to be the primary cause of the clinical presentation were included. Cases were excluded if any imaging findings were considered incidental or unrelated to the clinical presentation (such as incidental congenital vertebral body malformations in screw-tailed breeds), or if a concurrent lesion was identified outside of the T1–T6 region that was deemed to be responsible for the presenting clinical signs (i.e., in a dog with multifocal disease, also affecting the cranial thoracic region, but presenting for a cervical myelopathy). Descriptive statistical analysis and tables were performed with Microsoft Excel.

## Results

### Signalment

A total of 84 dogs met the inclusion criteria (Supplementary Table Sp1), consisting of 55 males (65%), of which 36 were neutered, and 29 females (35%), of which 19 were neutered. The breed distribution is summarised in Table 1 and included French Bulldog (*n* = 8), Labrador Retriever (*n* = 7), English Bulldog (*n* = 6), German Shepherd dog (*n* = 6), Golden Retriever (*n* = 5), Staffordshire Bull terrier (*n* = 5), Springer spaniel (*n* = 4), West Highland White terrier (*n* = 4), Dogue de Bordeaux (*n* = 3), Pug (*n* = 3), Boxer (*n* = 2), Cairn terrier (*n* = 2), Cocker spaniel (*n* = 2), Dachshund (*n* = 2), English Bull terrier (*n* = 2), Miniature Schnauzer (*n* = 2), Rottweiler (*n* = 2), and one of each of the following: Border Collie, Chihuahua, Clumber spaniel, Collie, Flat Coated Retriever, Gordon Setter, Great Dane, Irish Wolfhound, Italian Greyhound, Lurcher, Rhodesian Ridgeback, Schnauzer, and Tibetan terrier. Six dogs were classified as crossbreeds.

**Table 1:** Description and frequency of the different dog breeds.

**Breed**	**Cases (*n*)**
French Bulldog	8
Labrador Retriever	7
English Bulldog	6
German Shepherd dog	6
Crossbreed	6
Golden Retriever	5
Staffordshire Bull terrier	5
Springer Spaniel	4
West Highland White terrier	4
Dogue de Bordeaux	3
Pug	3
Boxer	2
Cairn terrier	2
Cocker Spaniel	2
Dachshund	2
English Bull terrier	2
Miniature Schnauzer	2
Rottweiler	2
Border Collie	1
Chihuahua	1
Clumber Spaniel	1
Collie	1
Flat Coated Retriever	1
Gordon Setter	1
Great Dane	1
Irish Wolfhound	1
Italian Greyhound	1
Lurcher	1
Rhodesian Ridgeback	1
Schnauzer	1
Tibetan terrier	1
**Total**	**84**

The mean age at diagnosis for all dogs was 81.1 months (standard deviation 53 months), with a median age of 87.5 months (range 2–205 months). Dogs with the presumptive (or confirmed) diagnosis of neoplasia had a median age at presentation of 120 months (range 57–205). Cases diagnosed with an anomalous aetiology had a median age at presentation of 6.5 months (range 2–125). The remainder of the disease aetiologies had a wider variation of ages within each group. Dogs in the group of degenerative diseases had a median age at diagnosis of 93.5 months (range 14–170). Inflammatory conditions presented at a median age of 48 months (range 7–136). Cases with infectious causes presented at a median age of 79 months (range 9–148). The single dog diagnosed with a vascular condition presented at 139 months of age. The age at presentation for the different disease processes is summarised in Table 2.

**Table 2:** Age at presentation for the different disease processes, presented in median and range (months).

**Disease process**	**Cases (*n*)**	**Median (months)**	**Range (months)**
Neoplasia	33	120	57–205
Anomalous	22	6.5	2–125
Degenerative	16	93.5	14–170
Inflammatory	7	48	7–136
Infectious	5	79	9–148
Vascular	1	139	N/A

N/A, not applicable.

### Onset

The onset of clinical signs was described in the clinical records to be acute in 43 dogs (51%) and chronic in 41 dogs (49%). When evaluating the different subsets of dogs based on the aetiology of the diagnosis, 18 dogs (55%) with neoplasia showed an acute onset and 15 dogs (45%) were considered to have a chronic onset. Anomalous and degenerative conditions were reported to show a chronic onset in 13 cases (59%) and 9 cases (56%), respectively. Inflammatory and infectious conditions presented with an acute onset in 5 (71%) and 3 (60%) cases, respectively. The one dog with a vascular disorder presented with an acute onset.

### Duration

The median duration of the clinical signs prior to presentation was 28 days, with a range from 1 to 1,825 days. The majority of cases (*n* = 40, 48%) had a duration of over 4 weeks, with an even distribution of cases having a duration of <2 weeks and from 2 to 4 weeks (*n* = 22 for each). The subset of dogs with a presumptive or final diagnosis of neoplasia showed an equal distribution of 11 cases (33%) for each of the three different groups of duration. Anomalous conditions frequently presented with a duration of over 4 weeks, seen in 15 cases (68%), while five cases (23%) had a 2–4 weeks duration and two cases (9%) presented with a <2 weeks duration of signs. A similar distribution was seen in dogs with degenerative aetiologies, with 11 dogs (69%) presenting with a duration of over 4 weeks, whilst three (19%) presented from 2 to 4 weeks and two (13%) showed a <2 weeks duration of signs prior to presentation. Inflammatory conditions more frequently presented within the first 2 weeks, comprising four dogs (57%) of this group, while two dogs (29%) presented with a history of 2–4 weeks and one dog (14%) had a history longer than 4 weeks. Cases with infectious conditions showed either a history of <2 weeks or over 4 weeks (40%, *n* = 2 for each), with one dog (20%) having a duration of clinical signs between 2 and 4 weeks. The one dog in the vascular group presented within the first 2 weeks.

### Progression

Most dogs (*n* = 76, 91%) showed progressive clinical signs prior to presentation, while seven dogs (8%) had static clinical signs, and one dog showed improving clinical signs. No dogs had a history of episodic clinical signs. All neoplasia cases (*n* = 33) showed worsening of the clinical signs prior to presentation. Seventeen dogs (77%) with anomalous disorders showed progressive clinical signs, whilst the clinical signs in four dogs (18%) were reported to be static, and in one (5%) were considered to have improved by the time of presentation. Degenerative conditions were considered to be progressive in 14 cases (88%), while two cases (12%) were classified as having static clinical signs. Inflammatory and infectious conditions were more frequently described as showing progressive clinical signs, seen in six (86%) and five (100%) cases, respectively. The one dog with a vascular condition also showed progressive clinical signs.

### Clinical examination

General clinical examination at presentation was unremarkable in 64 dogs (76%), while 20 dogs (24%) showed abnormalities, which included concurrent joint disease in eight (10%), pyrexia in seven (8%), lethargy in six (7%) and lymphadenomegaly in four dogs (5%). Upon review of the neurological examination findings, 64 dogs (76%) were ambulatory and 20 (24%) were considered non-ambulatory. Eighty-three percent of ambulatory dogs (*n* = 53) were considered to show ataxia on examination. The cutaneous trunci muscle reflex was abnormal in 20 dogs (24% of all dogs) and ranged from completely absent (*n* = 9) to a variety of levels of interruption, from T6 to L4 spinous process.

The clinical signs were asymmetric in half of the cases (*n* = 42, 50%), with the most severely affected side being the left and right side in equal proportion (*n* = 21, 25% for each).

Urinary and/or faecal incontinence was reported in 4% (*n* = 3) of the dogs; however, one dog showed chronic urinary incontinence secondary to urinary sphincter mechanism incompetence and was receiving oral estriol prior to the development of the myelopathy. One dog had urinary incontinence only and the third dog showed urinary and faecal incontinence. None of the dogs showed signs of Horner syndrome.

A short-strided thoracic limb gait was observed in 12 dogs (14%). All dogs with this feature also showed pelvic limb neurologic abnormalities, with seven dogs (58%) being ambulatory with pelvic limb ataxia, and five dogs (42%) showing non-ambulatory paraparesis/paraplegia. Supplementary Video is available showing variable degree of pelvic limb dysfunction in association with a short-strided thoracic limb gait. Signs of apparent discomfort on vertebral column palpation were observed in 46 cases (55%).

### Neuroanatomic localisation

The recorded neuroanatomic localisation was most commonly within the T3-L3 spinal cord segments (75%, *n* = 63), with 10 dogs (12%) having a neurological examination suggestive of a C6-T2 myelopathy and one dog (1%) suggestive of a C1-C5 myelopathy. Signs of apparent spinal hyperaesthesia without obvious neurological deficits were record in 12% (*n* = 10) of dogs. A summary of the frequency of the different neuroanatomic localisations can be found in Table 3.

**Table 3:** Frequency of the different neuroanatomic localisations.

**Neuroanatomic localisation**	**Cases (*n*)**
C1-C5	1
C6-T2	10
T3-L3	63
Spinal hyperaesthesia alone	10

### Advanced imaging

According to the study inclusion criteria, all selected cases had advanced imaging of the vertebral column that revealed an abnormality in the region of the T1-T6 vertebrae deemed to be responsible for the presenting clinical signs. Magnetic resonance imaging was used in 75 cases (89%) and CT was used in the remainder (*n* = 9). The lesion location pattern was characterised as extradural in 70 cases (83%), intramedullary in nine cases (11%), and intradural-extramedullary in five (6%).

### Final clinical diagnoses

A definitive diagnosis was achieved in 53 cases (63%) either by distinctive imaging features (i.e., congenital malformations, discospondylitis, intervertebral disc extrusion/protrusion) (*n* = 33), cytology following fine needle aspiration (FNA) of the lesion (*n* = 6), surgical excision/biopsy collection or post-mortem examination (*n* = 14). The most commonly observed disease process was neoplasia, diagnosed in 33 dogs (39%), followed by anomalous conditions in 22 dogs (26%), degenerative disorders in 16 dogs (19%), presumed inflammatory immune-mediated conditions in seven dogs (8%), infectious causes in five dogs (6%), and vascular disease in one dog (1%).

**Figure 1:**
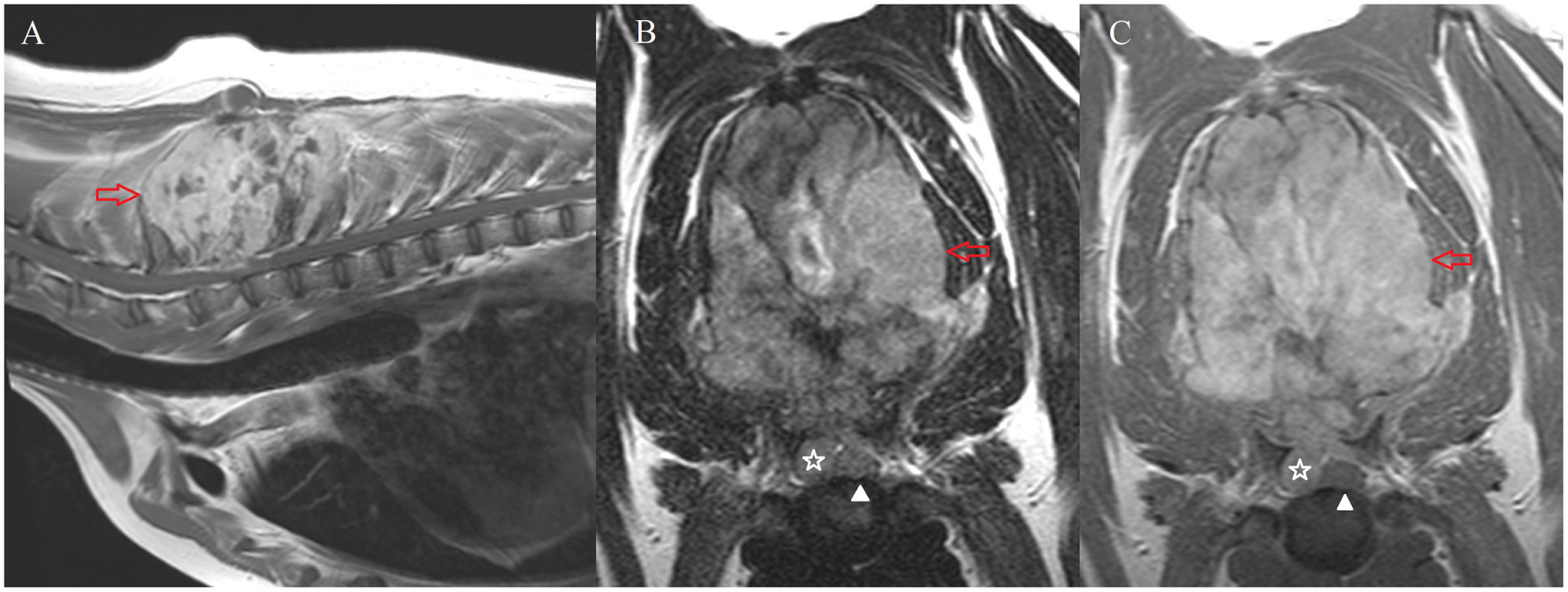
MRI of case #72. Sagittal T1-weighed post-contrast image of the caudal cervical and cranial thoracic vertebral column **(A)** transverse T2-weighted **(B)** and transverse T1-weighted post-contrast **(C)** images at the level of T1-T2. A large, irregular and well-defined mass lesion involving the dorsal spinous processes of T1-T4 vertebrae is shown (red arrow). This mass is markedly heterogeneous on T2-weighted images **(B)** and shows marked enhancement in T1-weighted images after intravenous administration of gadolinium-based contrast agent **(A,C)**. The mass showed extradural extension causing moderate spinal cord compression (star). The white arrowhead indicates the position of the spinal cord. On post-mortem examination, this mass was classified as a chondroblastic osteosarcoma.

Regarding the 33 cases of suspected or confirmed neoplasia, 17 (52%) were considered locally invasive, 10 (30%) were considered metastatic in the presence of other confirmed lesions, and six (18%) were considered primary CNS neoplasia. Most of the observed masses (70%, *n* = 23) were presumed to be of mesenchymal origin (e.g., Figure 1). Within this group of mesenchymal neoplasia, these were diagnosed as presumed sarcoma in 18 cases (78%) based on imaging features or confirmed with support of FNA cytology (*n* = 3) or post-mortem examination (*n* = 5). In the latter, diagnoses ranged from histiocytic sarcoma (*n* = 2), liposarcoma (*n* = 1), osteosarcoma (*n* = 1) and metastatic spindle-shaped mesenchymal neoplasia (*n* = 1). All but two cases of locally invasive neoplasia were characterised as “sarcoma” in origin, with the others being diagnosed as multiple myeloma (*n* = 1) and round cell tumour (*n* = 1). In the 10 cases (30%) of presumed or confirmed metastatic neoplasia, sarcoma-type tumours accounted for 8 cases (80%); other types included lymphoma (*n* = 1) and neuroendocrine carcinoma (*n* = 1). Suspected or confirmed primary CNS neoplasia included meningioma (*n* = 4) and primary central nervous system (CNS) lymphoma (*n* = 2).

Diagnoses observed in the 22 dogs with anomalous conditions included vertebral body malformations in 14 (64%), followed by cranial thoracic vertebral canal stenosis in six (27%) and the complex of spina bifida and dermoid sinus in two (9%). Most of the dogs diagnosed with vertebral body malformations were screw-tail breeds (64%, *n* = 9), consisting of French bulldogs (*n* = 6), Pugs (*n* = 2), and an English bulldog (*n* = 1), while other breeds included English bull terrier, Labrador retriever, Rhodesian ridgeback, Rottweiler, and German Shepherd dog (*n* = 1 of each). Cranial thoracic spinal stenosis was mostly observed in large breed dogs, being either Dogue de Bordeaux (*n* = 3), Great Dane (*n* = 1), or Rottweiler (*n* = 1). The complex of spina bifida and dermoid sinus was observed in retriever breeds, one Labrador and one Golden retriever.

**Figure 2:**
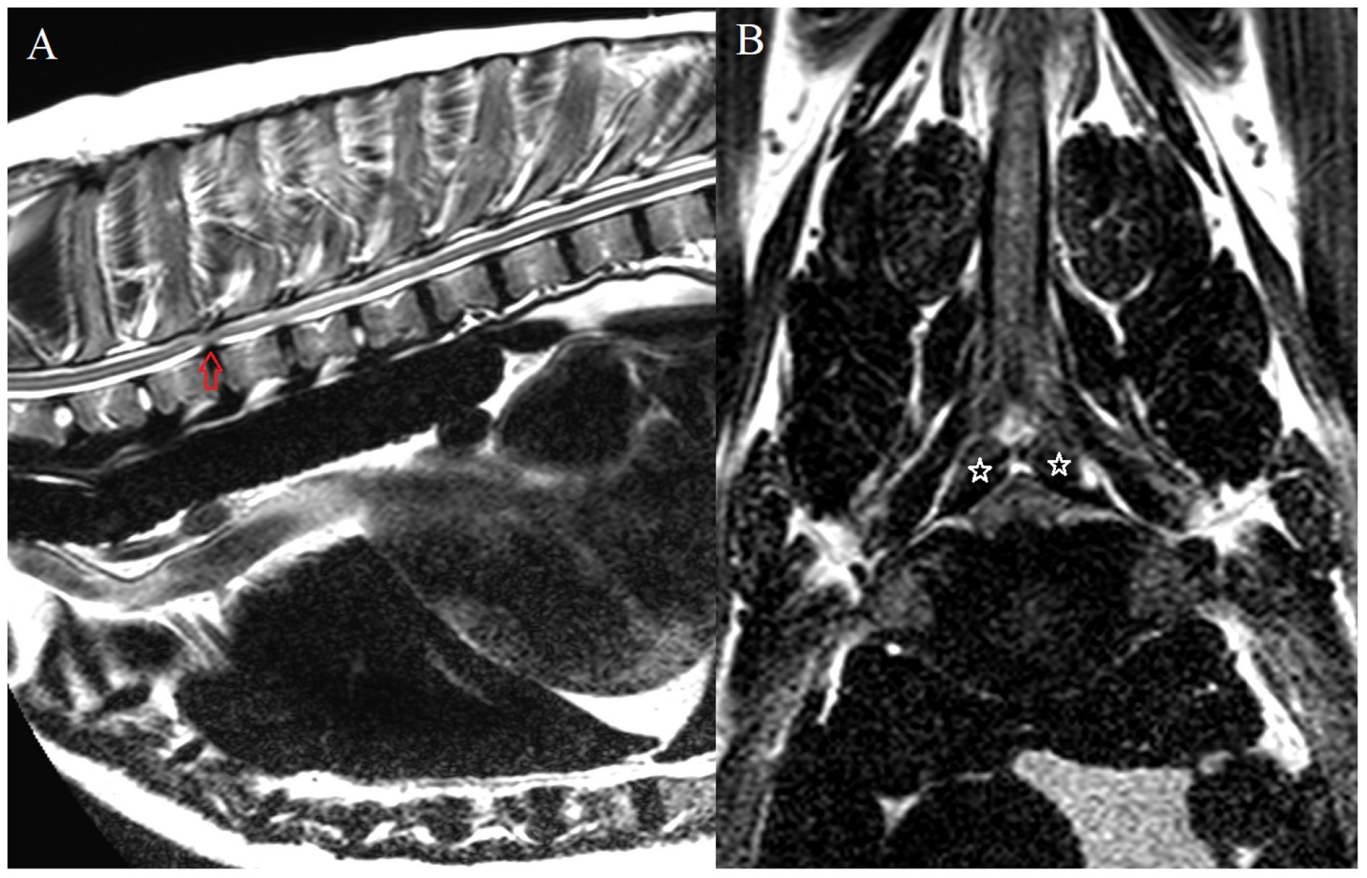
MRI of case #34. Sagittal T2-weighed image of the cranial thoracic vertebral column **(A)**; transverse T2-weighted image at the level of T2-T3 **(B)**. At this level (T2-T3), there is an intervertebral disc protrusion which resulted in moderate ventral spinal cord compression (red arrow). Additionally, there is apparent bilateral vertebral articular process hypertrophy, causing mild bilateral dorsolateral spinal cord compression (stars). This was confirmed upon surgical decompression (T2-T3 hemilaminectomy).

Degenerative conditions observed in the area of interest (T1-T6 vertebrae) included intervertebral disc protrusion (IVDP) in nine dogs (56%), intervertebral disc extrusion (IVDE) in four dogs (25%), juxta-articular cyst in two dogs (13%) and presumed acquired spinal arachnoid diverticulum in one dog (6%). The German Shepherd dog was the most represented breed in the IVDP group (*n* = 4, e.g., Figure 2), while the other breeds included English bulldog (*n* = 2), French bulldog, Pug and crossbreed (*n* = 1 for each breed). Regarding the diagnosis of IVDE, this occurred solely in chondrodystrophic breeds, which included Clumber spaniel, Cocker spaniel, Dachshund and Springer spaniel (n = 1 each).

All dogs in the group of inflammatory (suspected immune-mediated) myelopathies were diagnosed with meningomyelitis of unknown origin (MUO) based on advanced imaging findings, supported by cerebrospinal fluid (CSF) analysis (*n* = 7). The CSF results were normal in 2 dogs, showed albuminocytologic dissociation in one dog (total protein of 47.8 mg/dL, reference <45), marked predominantly neutrophilic pleocytosis in one dog (total nucleated cell count 2,070 cells/μL, reference <5) and marked mononuclear pleocytosis in one dog (total nucleated cell count 1,675 cells/μL, reference <5). The CSF was considered non-diagnostic due to marked blood contamination in one dog, and was reported to suggest an inflammatory process, without further information being available, in the final case. All dogs with an infectious origin for the clinical signs were diagnosed with discospondylitis. One dog was diagnosed with primary haematomyelia at the level of the T4 vertebral body, which accounted for the single case with a vascular cause for a T1-T6 myelopathy. Post-mortem examination and histopathology did not reveal any evidence of vascular malformation, neoplasia or another underlying cause for this lesion.

### Location

**Figure 3:**
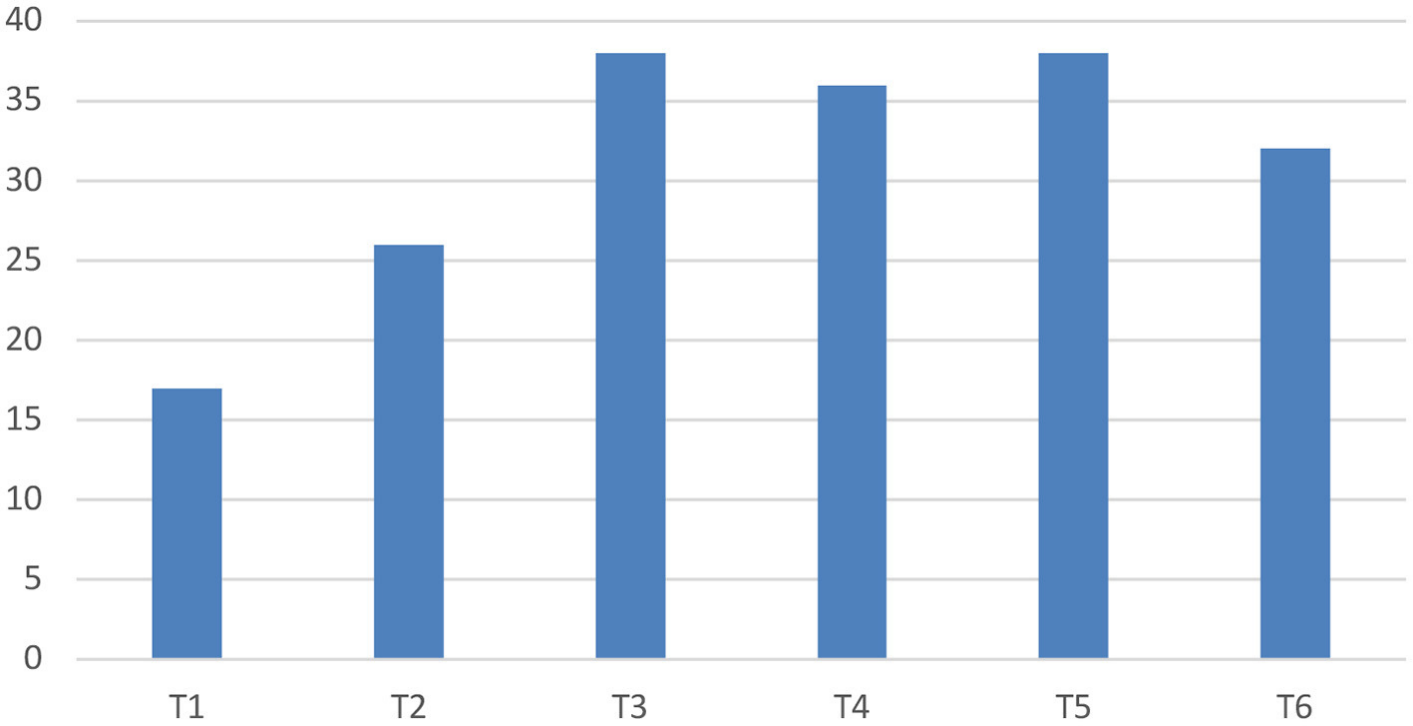
Lesion location if a single vertebral body is considered.

**Figure 4:**
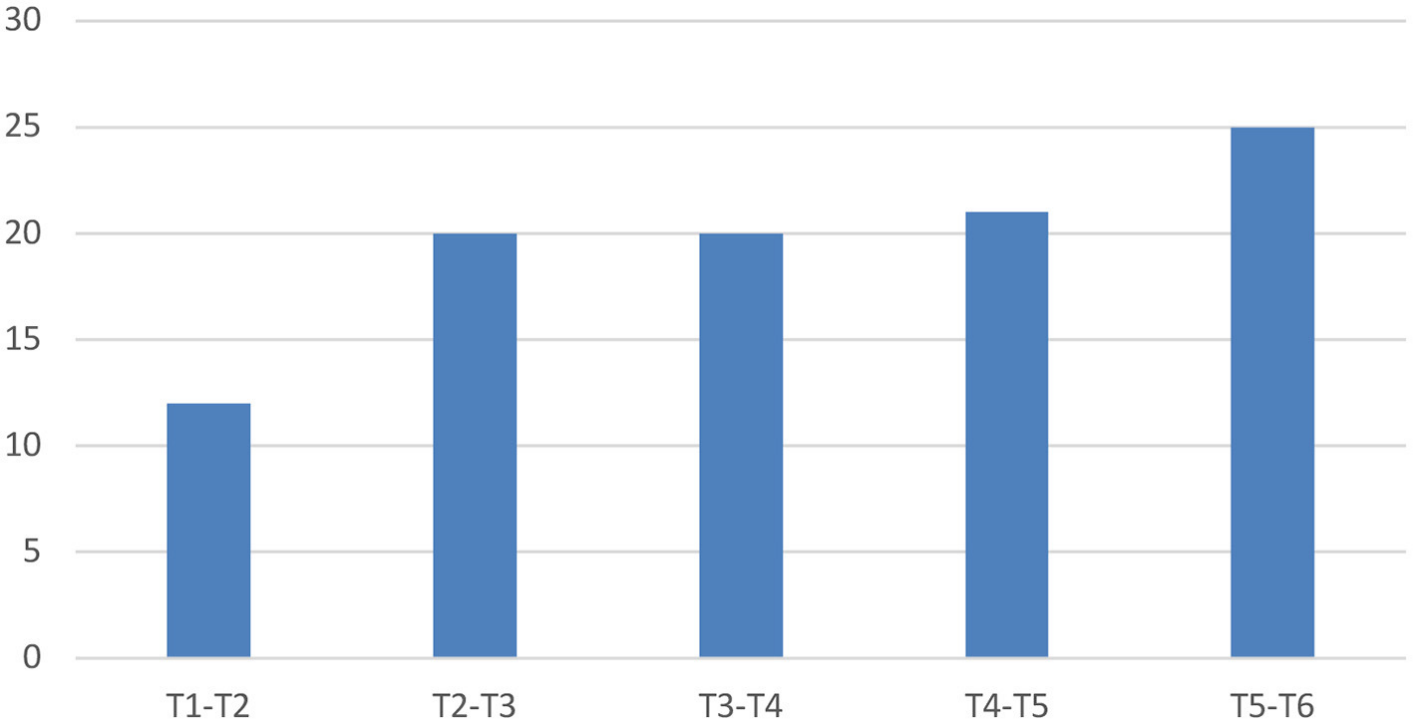
Lesion location if a block of two vertebrae and their intervertebral disc space is considered.

Within the area of interest of our study (T1-T6 vertebrae), lesions were most commonly observed at the level of T3 and T5 (*n* = 38 for each) followed by T4 (*n* = 36) (Figure 3). If a block of two vertebrae and their intervertebral disc space is considered, then the area of T5-T6 was the most affected region (*n* = 25), followed by T4-T5 (*n* = 21), and T2-T3 and T3-T4 (*n* = 20 for each) (Figure 4).

Neoplasia was most frequently detected in the area of T3-T4 (*n* = 8), closely followed by T2-T3 (*n* = 7) and T1-T2 (*n* = 6). If only a single vertebra was considered, then the T3 vertebra was the most affected (*n* = 17) followed by lesions affecting the T1, T4, and T6 vertebral bodies (*n* = 11 for each), with several cases having multiple affected vertebrae.

In dogs with anomalous conditions, the most frequently affected vertebrae were T5 (*n* = 13), T6 (*n* = 12), and T2 (*n* = 10). The T5-T6 intervertebral disc space was most commonly affected for these anomalous conditions (*n* = 11), followed by T2-T3 (*n* = 8) and T4-T5 (*n* = 7).

The most common site of compression for the cases with IVDP were T2-T3 and T5-T6 (*n* = 3 each), followed by T3-T4, T4-T5, and T6-T7 (*n* = 1 each). Regarding the IVDE cases, the most common area was the T4-T5 disc space (*n* = 2), followed by T1-T2 and T3-T4 (*n* = 1 for each).

Cases with suspected inflammatory spinal cord disease were most commonly seen over the T4-T5 intervertebral space and respective vertebral bodies (*n* = 6). The diagnoses of discospondylitis were observed at T5-T6 (*n* = 3), T4-T5 (*n* = 1), or T3-T4 (*n* = 1). As previously reported, the case of primary haematomyelia was identified over the T4 vertebra.

### Asymmetry of clinical signs, short-strided thoracic limb gait, and hyperaesthesia on spinal palpation between the different disease groups

When the proportion of dogs showing asymmetric neurologic deficits was considered between the different aetiologies, degenerative conditions frequently showed asymmetric clinical signs, seen in 15 dogs (94%) within this group. Cases of neoplasia showed asymmetry in 17 cases (52%), while the clinical signs of three dogs (43%) diagnosed with inflammatory CNS disease were asymmetric. The cases within the anomalous groups showed asymmetric clinical signs in seven dogs (32%). The single case in the vascular group showed symmetric clinical signs.

A short-strided thoracic limb gait was observed in 12 dogs (14%). The majority of these dogs were recorded as having a suspected T3-L3 myelopathy (83%, *n* = 10), with only 2/12 dogs having a suspected lesion within the C6-T2 spinal cord segments. Final diagnoses found in the dogs with a short-strided thoracic limb gait included neoplasia (67%, *n* = 8) and one case of degenerative, inflammatory, anomalous and vascular disease, respectively. The lesions in dogs with this particular feature were localised mostly within the T3-T4 vertebral bodies (*n* = 4), however could be seen as far caudal as the T5-T6 vertebral bodies (*n* = 2). The areas of the remaining cases included diffuse/multifocal disease (*n* = 3), T2-T3 (*n* = 2) and T1-T2 (*n* = 1). The three cases of diffuse / multifocal disease included concurrent T1 and T6 vertebral body lesions in one case, diffuse intramedullary spinal cord changes from T1 to T5 (suspected inflammation) in one case, and extensive extradural spinal cord compression from T1 to T4 in one case.

Regarding signs of apparent hyperaesthesia on examination, the majority of dogs with suspected neoplasia showed signs of hyperaesthesia on vertebral column palpation in the affected area (*n* = 23, 70%). Eleven dogs diagnosed with a degenerative condition showed no apparent discomfort on examination (69%), while the remaining five dogs (31%) were considered to show signs of pain. All dogs in the infectious (*n* = 5) and vascular (*n* = 1) groups showed apparent hyperaesthesia at presentation, with four out of five dogs with an infectious condition showing hyperaesthesia as the only clinical sign on examination. Regarding the dogs within the inflammatory group, all but one (*n* = 6, 86%) presented with apparent pain upon palpation of the vertebral column. Lastly, most dogs (*n* = 16, 73%) with anomalous diseases showed no apparent signs of discomfort.

## Discussion

This study describes the signalment, onset and progression prior to diagnosis, clinical examination findings, most commonly affected locations and presumptive or final diagnoses in 84 dogs presenting for a disease process affecting the cranial thoracic vertebral column. To the authors' knowledge, this is the first study to focus on lesions affecting this specific region, and the information regarding the most common presenting signs and different aetiologies responsible for these signs will assist with clinical decision making in practise.

Considering the whole pool of clinical cases described in our study, the most frequent disease process causing a cranial thoracic myelopathy was neoplasia. These dogs had a median age of 120 months (range 57–205) at the time of presentation, had either an acute (55%) or chronic (45%) onset, showed progressive clinical signs in all cases, and the majority had apparent discomfort on clinical examination (70%). The underlying origin of the neoplasia was variable, with most cases considered to show imaging features of sarcoma (78%). This is a broad term describing malignant tumours of mesenchymal origin, commonly affecting bone, joints or muscle tissues (40). Comparison of these findings with the current literature on neoplasia affecting this region of the vertebral column is challenging as there are only scarce case reports or sporadic cases within larger studies. However, most described cases are locally invasive neoplasia [chondrosarcoma (34), multiple myeloma (36), myxosarcoma (37), nerve sheath tumour (35)], together with less than a handful of cases with primary CNS neoplasia [meningioma (13, 35)]. A recent study by Auger et al. (41) described the imaging features of extradural spinal neoplasia in 60 dogs. The exact location of the tumours was not described; however, the most affected region was the area of the T3-L3 vertebrae. The most common extradural neoplasia class was that of mesenchymal origin, accounting for 48% of all extradural spinal tumours (41). In the current study, suspected or confirmed mesenchymal tumours also accounted for the majority of cases within the neoplasia group (~70%). Regarding metastatic neoplasia, this was seen in 30% (*n* = 10) of our neoplasia cases, with the majority also being characterised as mesenchymal in origin considering either imaging features, fine needle aspirate samples or post-mortem evaluation. Only two of these metastatic cases were not considered to have a mesenchymal origin, being one round cell tumour (lymphoma) and one tumour of epithelial origin (neuroendocrine carcinoma). This finding is in contrast to previous reports which suggest carcinomas as the most common type of extradural metastasis in dogs (41, 42). The difference in prevalence of the different types of tumours between studies is interesting. However, it appears less likely that certain types of metastatic neoplasia may selectively affect different regions of the vertebral column, and these results may simply reflect small sample sizes, differences in the populations between our dataset and those included in the previous reports, or the lack of a confirmed histopathologic diagnosis in most of our cases characterised as suspected sarcomas.

The second most frequent aetiology observed in our study was anomalous disease, which describes a group of disorders with a developmental or congenital origin. These dogs had a median age of 6.5 months at presentation (range 2–125), showing a chronic onset (59%), and progressive clinical signs (77%) that had been present for over 4 weeks (68%). Spinal hyperaesthesia was an uncommon finding on examination of these patients (27%). The most common diagnosis observed was vertebral body malformation, particularly hemivertebrae with associated spinal cord compression that was seen in 14 of the 22 dogs diagnosed with an anomaly. The majority of these dogs were screw-tailed breeds (*n* = 9), which is similar to previous reports of vertebral body malformation where these breeds were also over-represented (26, 43). A typical breed distribution was observed in dogs diagnosed with cranial thoracic spinal stenosis, where most of the affected dogs were large breed Molosser-type dogs (Dogue de Bordeaux, Great Dane and Rottweiler) as previously described (20, 21).

Regarding degenerative conditions causing a cranial thoracic myelopathy, the cases in this subset had a median age at presentation of 93.5 months (range 14–170), showed progressive (88%), asymmetric (94%) and non-painful (69%) clinical signs, that had been present for over 4 weeks (69%) prior to presentation. The most common diagnosis for this disease category was IVDP, accounting for 56% of cases, followed by IVDE in 25% of cases. Similar to two other previous studies, our data also showed an over-representation of German Shepherd dogs with intervertebral disc disease within the cranial thoracic vertebral column (15, 16). Interestingly, Hearon et al. (16) did not identify any small-breed dogs presenting with intervertebral disc disease in the region of T1-T9, while our study identified intervertebral disc extrusions in several of the typical small chondrodystrophic breeds such as the Dachshund and Spaniel. These previous studies describe T2-T3 (15, 16), T3-T4 (15), and T4-T5 (15, 16) as the most common sites of intervertebral disc disease in the cranial thoracic region. The most common sites of compression for the cases in our study were T2-T3 and T5-T6 for IVDP, each accounting for 38% of cases (*n* = 3 for each), and T4-5 for IVDE (40%, *n* = 2). Despite this tendency, these sample sizes are too small to make any meaningful conclusions regarding the most common sites for intervertebral disc disease in this region.

Inflammatory, infectious and vascular disorders accounted for only a small portion of the 84 cases of our study, with the diagnoses of MUO (*n* = 7), discospondylitis (*n* = 5) and primary haematomyelia (*n* = 1), respectively. The cases in these categories were commonly acute in onset, seen in 71% of inflammatory and 60% of infectious cases, as well as in the one case of vascular origin. The age at presentation was similar in dogs with both inflammatory and infectious causes, affecting mostly middle-aged dogs (median of 48 months with range of 7–136, and median of 79 months with range of 9–148, respectively). The duration of clinical signs was most commonly <2 weeks, seen in 57% of inflammatory cases and in the dog with primary haematomyelia. The infectious disease group showed a uniform distribution of the duration of clinical signs between <2, 2 to 4, and more than 4 weeks. In all three of these disease categories, most dogs showed progression of clinical signs, with only one dog diagnosed with MUO showing a static presentation. It should be noted that there were no cases diagnosed with an ischaemic myelopathy affecting the cranial thoracic region, which is a more common cause of vascular spinal cord disease in dogs compared to haematomyelia and other haemorrhagic disorders (1). The typical clinical presentation for a dog with ischaemic myelopathy would include an acute or per-acute onset of a non-painful, often asymmetric myelopathy, with a static or improving disease course (44–46). This is a very different presentation of the progressive signs seen in the case with primary haematomyelia in this study. The T3-L3 spinal cord segments is the most commonly reported neuroanatomic localisation in dogs with ischaemic myelopathy (46, 47), and the absence of this common diagnosis within the cranial thoracic region is an interesting feature of this study. This may reflect the study's sample size and population, but it could also be hypothesised that this region is less commonly affected by ischaemic disorders in dogs, such as fibrocartilaginous embolism, compared to other areas of the spinal cord. This is supported by the fact that fibrocartilaginous embolism has previously been reported to most commonly affect the T9 to L3 region in dogs (48).

As expected, considering the study's region of interest (T1-T6 vertebrae), the most common neuroanatomic localisation following examination was the T3-L3 spinal cord segments (75%), with a small number of dogs showing clinical signs suggestive of a C6-T2 myelopathy (12%). Interestingly, one dog was presumed to have a C1-C5 myelopathy, mostly due to the presence of apparent discomfort on passive movement of the neck, in addition to mild pelvic limb ataxia and unilateral thoracic limb proprioceptive deficits; this dog was diagnosed with a T1-T2 intervertebral disc extrusion.

An interesting feature occasionally seen in some of our cases was the apparent gait abnormalities affecting all limbs, characterised by a short-strided thoracic limb gait in conjunction with paraparesis and pelvic limb ataxia, or paraplegia in some cases. This particular type of gait, often named a “two-engine” or “disconnected” gait when seen in ambulatory dogs, is frequently associated with pathology affecting the cervicothoracic (C6-T2) spinal cord segments and could therefore be expected to occur in association with lesions occurring within the mid-C5 to the caudal T1 vertebral bodies (3, 4, 49). This “two-engine” gait was observed in 12 dogs (14%) in our study, and most of these dogs (83%, *n* = 10) were considered to have a neuroanatomic localisation consistent with a T3-L3 myelopathy, due to normal postural reactions and segmental spinal reflexes in both thoracic limbs despite this characteristic gait. This is an interesting finding considering the small overlap of the anatomical area usually associated with this gait (up to caudal T1 vertebra) and the area of interest in our study (T1-T6 vertebrae). In fact, all but one of the dogs reported with this gait change were diagnosed with a lesion caudal to the T1 vertebra; the most commonly affected area being T3-T4, and some lesions as far caudal as the T5-T6 vertebral bodies. This short-strided thoracic limb gait in conjunction with pelvic limb neurological deficits has been previously anecdotally reported in dogs with conditions affecting the cranial thoracic vertebral column, and has been suggested to be caused by a local focus of pain (i.e., due to osseous neoplasia) rather than reflecting a true neurological deficit (i.e., thoracic limb paresis) as for cases with a C6-T2 myelopathy (4). As a result, these patients show normal thoracic limb flexor withdrawal reflexes and hopping responses (4). However, half of the cases with a “two-engine” gait in this study were not reported to show apparent discomfort on examination. Neoplasia was the most common diagnosis in dogs with this gait abnormality at presentation, diagnosed in two thirds of the cases (*n* = 8), with the other diagnoses being one case of each of the following: intervertebral disc protrusion (degenerative), meningomyelitis of unknown origin (inflammatory), cranial thoracic spinal stenosis (anomalous) and haematomyelia (vascular). In the cases with no apparent discomfort on examination, it is uncertain why these patients presented with this gait abnormality. However, it is possible that it was still related with a focus of discomfort in a stoic dog, particularly as the cranial thoracic vertebral bodies and spinal canal lie deep to the skin surface in this area, and foci of discomfort may therefore be challenging to identify compared to other parts of the vertebral column.

Another possibility for the presence of the “two-engine” gait in cases with cranial thoracic lesions may be a variation of the contribution of the spinal nerve roots to the brachial plexus. Previous anatomic studies in dogs have found four different patterns of segmental innervation of the brachial plexus, described as C6-T1, C5-T1, C6-T2, and C5-T2, depending on the location of the nerve root contributions (50). Subsequent studies using electrophysiology to determine spinal nerve root contributions to the different nerves of the brachial plexus, grouped the nerve roots to the median, ulnar and musculocutaneous nerves into C6-T2 or C6-T1 patterns, and to the radial nerve into C6-T1, C6-T2, and C7-T2 groups (51, 52). Variation in nerve root contribution to the brachial plexus has been described in human medicine as *pre-fixed* or *post-fixed* brachial plexus, which represent a more cephalic or caudal contribution, respectively (53). This variation in nerve root contribution has been documented in the lumbosacral plexus in dogs but was not apparent in the brachial plexus (52, 54). Considering the available literature in dogs, brachial plexus contribution has not been identified caudal to T2 (51, 52). Thus, there is lack of support for the possibility of a post-fixed brachial plexus, with more caudal contributions, as possible explanation of the “two-engine” gait in dogs with cranial thoracic myelopathies, and additional anatomic studies would be required to further assess this hypothesis.

Urinary and/or faecal incontinence was scarcely seen within our population. This was documented in three cases, however only two of those were presumed to be secondary to the cranial thoracic myelopathy, which were still ambulatory despite the urinary/faecal incontinence. A myelopathy in the area of interest of our study, from T1 to T6, could cause a supra-sacral (cranial to the L7 spinal cord segment) incontinence (55). This can occur due to the damage of the upper motor neuron pathways to the bladder and rectum, resulting in a retention urinary incontinence, due to increased sphincter tone, and faecal incontinence due to the inability to control defecation once the defecation reflex has been triggered (55). These signs are more commonly observed in cases with severe dysfunction of these suprasacral spinal cord segments, resulting in tetra-/paraplegia. However, it can occasionally be seen in animals that remain ambulatory (3). Faecal incontinence in animals that retain the ability to walk can occur particularly if the disease process is involving the dorsal aspect of the spinal cord, causing disruption of the sensory pathways (56), which was suspected to explain the faecal incontinence observed in one dog of this study.

There are several limitations to this study, many of which are inherent to any retrospective data analysis. The main limitation was the lack of a histopathologic definitive diagnosis in the majority of the cases, with most cases achieving a presumptive diagnosis based on advanced imaging interpretation and ancillary tests (e.g., CSF analysis, FNA cytology). Magnetic resonance imaging has a reported good sensitivity (86.8%) but only moderate specificity (64.7–72.5%) for the diagnoses of spinal neoplasia, and good sensitivity (81.5%) for the classification of inflammatory spinal cord diseases when clinical data is also provided (57, 58). A second limitation is that a small number of dogs (*n* = 9) had CT rather than MRI performed. However, both CT and MRI are still considered valuable techniques to diagnose disorders affecting the spinal cord (57). Other limitations, also related to the retrospective nature of the study, include the classification of the rate of onset as either acute or chronic based on the terminology used and description available in the clinical records, rather than being more precisely defined according to the number of hours or days over which the signs appeared. The lack of precise clinical information available in some records, particularly regarding the specific rate of onset, duration and progression of the clinical signs may result in ambiguous data, which should be interpreted with caution. Additionally, the retrospective classification of the different diagnoses within the VITAMIN-D mnemonic can often be debatable, as some diagnoses may fall under different classifications depending on the dog's signalment at the time of presentation (i.e., spinal arachnoid diverticula or cranial thoracic spinal stenosis), which may have slightly skewed the descriptive analysis. Finally, despite the reasonable overall sample size when compared to other similar studies in veterinary medicine, some of the individual group sizes were still small, of which three disease groups (inflammatory, infectious and vascular) had <10 cases each.

In conclusion, this study describes the signalment, clinical presentation and final diagnoses observed in dogs with evidence of a cranial thoracic (T1-T6) lesion on advanced imaging that explains the presenting neurological signs. The most common diagnosis was neoplasia (39%), closely followed by anomalous (26%) and degenerative (19%) disorders. Most dogs underwent MRI and the most common vertebrae affected were T3 and T5. The majority of dogs presented with a progressive history of over four-weeks duration. On examination, most dogs were ambulatory and despite the most common neuroanatomic localisation being the T3-L3 spinal cord segments, ~14% (12/84) of dogs showed a short-strided thoracic limb gait. Dogs with degenerative conditions commonly showed asymmetric clinical signs, and the majority of dogs with neoplasia showed signs of spinal hyperaesthesia on examination. The findings of this study can assist with the clinical reasoning when managing such cases in practise.

## Data availability statement

The original contributions presented in the study are included in the article/Supplementary material, further inquiries can be directed to the corresponding author.

## Ethics statement

The animal study was reviewed and approved by University of Glasgow School of Veterinary Medicine Research Ethics Committee. Written informed consent was obtained from the owners for the participation of their animals in this study.

## Author contributions

DS-M and EI were responsible for the study concept. Retrospective data collection was performed mostly by BL, in cooperation with RJ-L, RG-Q, JA, and PF. Statistical analysis was mostly performed by BL with the cooperation of JR. BL was responsible for the writing of the manuscript. All authors reviewed and contributed to the article and approved the submitted version.

## Funding

Linnaeus Veterinary Limited supported the costs of the Open Access Publication Charges.

## Conflict of interest

Authors BL, EI, and DS-M were employed by Linnaeus Veterinary Limited. The remaining authors declare that the research was conducted in the absence of any commercial or financial relationships that could be construed as a potential conflict of interest.

## Publisher's note

All claims expressed in this article are solely those of the authors and do not necessarily represent those of their affiliated organizations, or those of the publisher, the editors and the reviewers. Any product that may be evaluated in this article, or claim that may be made by its manufacturer, is not guaranteed or endorsed by the publisher.
